# Synergic Effect of Isolated Ce^3+^ and Pt^δ+^ Species in UiO-66(Ce) for Heterogeneous Catalysis

**DOI:** 10.1021/acscatal.3c00502

**Published:** 2023-06-26

**Authors:** Sergio Rojas-Buzo, Benjamin Bohigues, Davide Salusso, Avelino Corma, Manuel Moliner, Silvia Bordiga

**Affiliations:** † Department of Chemistry and NIS Centre, University of Turin, Via Giuria 7, 10125 Turin, Italy; ‡ Instituto de Tecnología Química, Universitat Politècnica de València-Consejo Superior de Investigaciones Científicas, Avenida de los Naranjos s/n, 46022 València, Spain; § European Synchrotron Radiation Facility, CS 40220, 38043 Cedex 9 Grenoble, France

**Keywords:** Ce-MOF, Ce^3+^, electrostatic deposition, Pt single site, CO oxidation

## Abstract

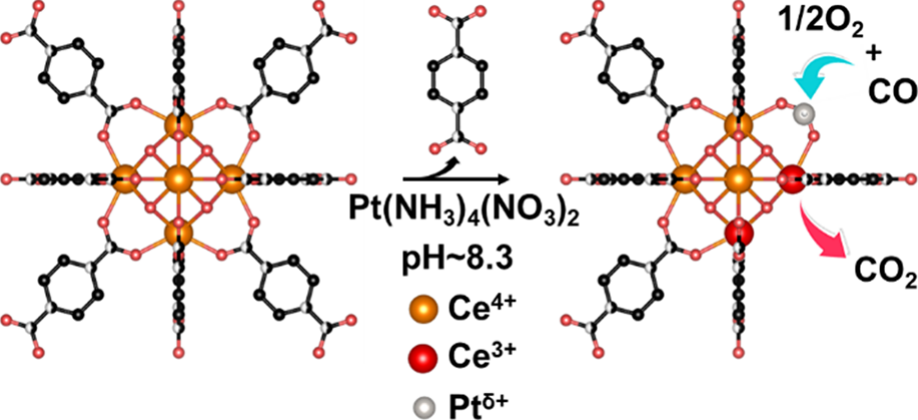

In this work, we have synthesized through an efficient electrostatic deposition a Pt single-atom catalyst (SAC) supported on a Ce-MOF. The basic solution employed in the impregnation process favors the deprotonation of the hydroxyl groups allocated on the clusters that can easily interact with the cationic Pt species. The resulting material, denoted as Pt/UiO-66(Ce), shows an increment of Ce^3+^ content, as demonstrated by UV–vis and Ce L_3_-edge XANES spectroscopy. These Ce^3+^ species and their corresponding oxygen vacancies are able to accommodate very disperse Pt single sites. Moreover, Pt L_3_-edge XANES and CO-FTIR spectroscopy confirm the cationic nature of the supported Pt^δ+^ (2+ < δ < 4+). For comparison purpose, we have synthesized and characterized a well-known Pt single-site catalyst supported on nanocrystalline ceria, denoted as Pt/*n*CeO_2_. Since the simultaneous presence of Ce^3+^ and Pt^δ+^ on the MOF clusters were able to activate the oxygen molecules and the CO molecule, respectively, we tested Pt/UiO-66(Ce) for the CO oxidation reaction. Interestingly, this catalyst showed ∼six-fold increment in activity in comparison with the traditional Pt/*n*CeO_2_ material. Finally, the characterization after catalysis reveals that the Pt nature is preserved and that the activity is maintained during 14 h at 100 °C without any evidence of deactivation.

## Introduction

Although noble metals, such as Pt, Pd, Rh, Ir, Ru, and Au, are some of the least abundant elements on earth, their distinctive catalytic properties offer unique performances in many chemical processes, particularly those related to energy and environmental fields, such as oil refining, control vehicle emissions, or in fuel cells, among others.^1−3^ For this reason, many recent research efforts are devoted to develop more efficient noble metal-based catalysts, both in terms of catalytic performance (activity and selectivity) and stability, while better rationalizing the use of the requested metallic sources and maximizing the atom economy.^4−8^ In this sense, single-atom catalysts (SACs) with isolated metal atoms dispersed on solid supports have shown significant relevance in heterogeneous catalysis in the last decade.^3,9−12^ The reason is because SACs traditionally present uniformly distributed and well-defined supported active sites, approaching the unique active site definition achieved by homogeneous catalysts,^13−15^ fact that combined with their maximized metal atomic efficiency and unique electronic properties, offer them enhanced activities and selectivities compared to classical heterogeneous catalysts in a wide range of chemical reactions.^3,16,17^ However, leaching of active species into liquid-phase reactions and metal sintering through Ostwald ripening are common deactivation pathways of SACs, and, consequently, rationalizing the preparation of highly stable single atoms through strong metal–support interactions is a very challenging task today.^14,18,19^

SACs have specifically demonstrated excellent catalytic performances for low-temperature reactions,^14^ such as low-temperature CO oxidation.^14,20,21^ Low-temperature CO oxidation is a very important reaction to improve the control of automobile emission, the purification of gas streams derived from petrochemical industry, and pure hydrogen production for proton-exchange membrane fuel cells.^22−24^ Among the different SACs described in the open literature for CO oxidation, single Pt and Pd atoms deposited over CeO_2_-based catalytic systems have exhibited remarkable and improved low-temperature activities for CO oxidation reactions.^20,23,25^ This enhanced CO oxidation activity has been correlated with the specific structural and electronic properties of the ceria support, which provide not only excellent oxygen mobility but also strong metal–ceria stabilization.^20,23,25,26^ Indeed, the ability to maximize the reactive surface of the ceria support by decreasing the particle size into the nanoscale, i.e., 8–10 nm, has been demonstrated as an excellent strategy to maximize the metal–support interactions, permitting, at the same time, the enhancement of the catalytic performance for the CO oxidation reaction.^25,26^

Metal–organic Frameworks (MOFs) have been considered as excellent support candidates to stabilize single atoms thanks to their unique metal–support interactions. These can be adequately tuned by controlling the nature of the organic functional ligands and/or metal oxoclusters employed in their preparation as well as their elevated surface area that allows introducing high metal dispersions.^27,28^ Among the different synthesis strategies reported in the literature to obtain MOF-stabilized SACs, the so-called metal node stabilization strategy is a very interesting approach. This methodology permits anchoring the target isolated single atoms onto the subnanometric metal oxoclusters, inducing unique electronic metal–support interactions with very distinctive properties compared to SACs supported in bulk supports.^27−30^ However, regarding the stability of the metal–support interaction, it is also important to consider separately the nature of the metal and MOF. For example, Xue et al. have reported the different stabilities of single-atom metal ions supported on Zr metal clusters.^31^ They found that Pt^2+^ shows the highest stability with respect to other divalent metals anchored on the nodes. On the other hand, it is worth noting that this metal node stabilization strategy requires the selected MOF acting as the support to show coordinately unsaturated node sites to host the metallic single atoms that will be postsynthetically incorporated.^32^

Nanoparticulate ceria with sizes ∼8−10 nm has been described as a superb support to design low-temperature active and stable SACs.^20,23^ In this sense, Ce-based MOFs presenting subnanometric CeO_2_ clusters of a few Ce atoms could potentially even enhance the single-atom metal–support interactions achieved by the nanoparticulate ceria. In particular, UiO-66(Ce) MOF presents hexanuclear Ce_6_(μ_3_-O)_4_(μ_3_-OH)_4_ nodes connected by linear 1,4-benzenedicarboxylic acid linkers,^33^ with a very large amount of accessible nodes containing Ce^3+^ defect sites (∼50% of Ce_6_ nodes contain at least one Ce^3+^ atom),^34,35^ which could be ideal anchoring sites to stabilize single atoms.^36^

With this in mind, herein, we have rationalized the preparation of a single-atom Pt catalyst supported on a Ce-based MOF, Pt/UiO-66(Ce), by following a simple and efficient strong electrostatic adsorption strategy on UiO-66(Ce). Different characterization techniques allow unraveling the isolated platinum atoms that are strongly coupled to the oxygen vacancies of subnanometric cerium oxide units of a defective UiO-66(Ce). To demonstrate the special properties of a SAC Pt/UiO-66(Ce) material, a well-defined Pt/nanoceria (Pt/*n*CeO_2_) has been prepared and characterized for comparison purposes. A novel SAC Pt/UiO-66(Ce) shows an improved catalytic performance per active site for the low-temperature CO oxidation reaction compared to Pt/*n*CeO_2_.

## Materials and Methods

### Sample Preparation

#### Synthesis of UiO-66(Ce)

This synthesis has been carried out following a scaled-up previously reported recipe:^33^ H_2_BDC (1,4-benzenedicarboxylic acid) (3.82 g, 23 mmol) and DMF (121 mL) were placed in a round-bottom flask. Then, an aqueous solution of cerium ammonium nitrate (43 g, 0.53 M) was added to the mixture. The flask with the resulting solution was sealed and heated under magnetic stirring for 15 min at 100 °C. The as-obtained yellow precipitate was decanted by centrifugation from the mother solution and was washed three times with DMF and three times with acetone.

#### Preparation of the Supported Pt/UiO-66(Ce)

The single-atom Pt catalyst deposited on UiO-66(Ce) was prepared by the basic wet impregnation method, using sodium bicarbonate as the basic component. First, 1.0 mg of Pt(NH_3_)_4_(NO_3_)_2_ and 24.0 mg of NaHCO_3_ were dissolved in 10 mL of deionized water. Then, 0.5 g of UiO-66(Ce) was added in the resultant mixture and stirred at room temperature for 14 h. The obtained solid, denoted as Pt/UiO-66(Ce), was filtered and washed with deionized water.

#### Preparation of the Supported Pt/*n*CeO_2_

This material has been prepared by incipient wetness impregnation according to the previously reported procedure:^37^ 63 mg of an aqueous solution of chloroplatinic acid (8%wt) was added dropwise to 0.6 g of *n*CeO_2_ (Rhodia). Then, the mixture was dried at 100 °C for 2 h. The resulting Pt/*n*CeO_2_ sample was calcined at 800 °C (1 °C/min) for 10 h in flowing air to obtain a high Pt dispersion.

#### CO Oxidation Reaction

In a typical experiment, 150 mg of each catalyst was mixed with 0.75 g of silicon carbide (Fischer Chemical) and loaded in a conventional tubular plug-flow reactor (ID = 9 mm). The reactor was heated at 100 °C during 10 min by passing a flow of N_2_ through the catalyst (50 mL/min) to eliminate the physisorbed molecules. Then, the temperature was lowered to 60 °C, and the reaction mixture was flowed at atmospheric pressure: 50 and 25 mL/min (depending on the selected gas-hourly space velocity: GHSV = 20,000 or 10,000 mL/g_cat_·h, respectively), containing 0.6% CO and 9.6% O_2_. Alternatively, the CO oxidation reaction has also been carried out under more diluted CO conditions: 55 mL/min (GHSV = 22,000 mL/g_cat_·h), containing 0.08% CO and 4.0% O_2_. The reaction temperature was varied from 60 to 100 °C. The downstream reaction effluents were analyzed continuously by gas chromatography.

### Sample Characterization

The PXRD measurements were carried out using the Bragg–Brentano geometry with a PANalytical PW3050/60 X’Pert PRO MPD diffractometer with a Cu anode (Kα = 1.5418 Å) and an X’Celerator detector.

The morphology of the samples was studied by field emission scanning electron microscopy (FESEM) using a ZEISS Ultra-55 microscope. The sample was placed on a carbon tape stuck on aluminum stubs.

Isothermal N_2_ physisorption measurements at liquid nitrogen temperature (LNT) were performed on a Micromeritics 3Flex. Prior to the measurement, the powders were degassed overnight at 120 °C. Specific surface areas using the Brunauer–Emmett–Teller (BET) model were calculated following the Rouquerol criterion.

Thermogravimetry analysis (TGA) data were recorded with a TA Instruments Q600 thermobalance in air flow (100 mL/min) with a ramp of 5 °C/min from RT to 500 °C, working with about 5 mg of sample in an alumina crucible.

Zeta-potential measurements (Zetasizer Nano ZS90 Malvern Instruments Ltd.) were conducted in aqueous media at different pH values. The sample was added to 10 mL of deionized water previously adjusted to the desired pH (2.5, 3, 3.5, 4.5, 5.5, 6.5, 8.5, and 10) with HCl or NaOH, sonicated for 1 min, and equilibrated for 5 min before measuring the zeta-potential. Each experiment was performed three times for each sample, and data are presented as mean ± standard deviation.

ICP analyses were carried out on a Varian 715-ES ICP-optical emission spectrometer after solid dissolution in H_2_SO_4_/H_2_O_2_ aqueous solution.

FT-IR spectroscopy in transmission mode was employed to characterize the surface properties of materials by following the adsorption of CO and CD_3_CN as probe molecules. Absorption/transmission FTIR spectra were collected using a Bruker Vertex 70 spectrophotometer equipped with a mercury cadmium telluride (MCT) cryodetector in the 4000–600 cm^–1^ range with 2 cm^–1^ resolution. Powders were pressed in self-supporting disks (∼4 mg/cm^2^) and placed in quartz IR cells suitable for thermal treatments in a controlled atmosphere and for spectral recording even at LNT. The heating experiment discussed in the study was performed with the following protocol: as soon as the temperature was stabilized at nominal 100 K, the liquid nitrogen reservoir was not refilled anymore. In this way, the temperature naturally increased with the consumption of liquid nitrogen until reaching RT. The temperature was qualitatively monitored from the IR spectra, i.e., with the increase of the temperature, the spectral transmittance decreased. When a stable variation of the transmittance was reached, the pellet approached room temperature (RT). The RT spectra were then collected after 30 min of stability in the spectral transmittance. Before IR measurements, catalysts were activated from RT to 110 °C at 5 °C/min holding at 110 °C for 2 h under dynamic vacuum. The spectra were treated using Bruker OPUS spectroscopy software. All the reported spectra were normalized for the pellet weight and area.

Diffuse reflectance (DR) UV–vis spectra were measured with a Varian Cary5000 spectrophotometer, equipped with a diffuse reflectance sphere. The samples were measured as-prepared. The spectra were collected in a reflectance mode and successively converted as Kubelka–Munk F(R) functions.

XPS spectra were measured using a SPECS spectrometer equipped with a 150 MCD-9 detector and a nonmonochromatic MgKα (1253.6 eV) X-ray source. The spectra were recorded with a 30 eV analyzer pass, an X-ray power of 50 W, under an operating pressure of 10^–9^ mbar. During data processing of the XPS spectra, binding energy (BE) values were referenced to the C 1s peak (284.8 eV). Spectral treatment has been performed using the CASA software. Ce (3d) peaks were fit using six and four Gaussian–Lorentzian (50:50) functions for Ce^4+^ and Ce^3+^ species, respectively. Spin–orbit splitting (Δ_s-o_ = 18.5 eV) was used to constrain the peak positions, while spin–orbit couples were forced to the same FWHM values. Spline Shirley function was employed to describe the background.

X-ray absorption spectra were measured at the BM23 beamline of the ESRF synchrotron facility. Transmission Ce L_3_-edge spectra were recorded with three ionization chambers. Two ionization chambers (IC) were filled with N_2_/He mixture for measuring the incoming beam (I_0_, 0.44 bar N_2_) and transmitted beam (I_1_, 1.49 bar N_2_), while a third IC (I_2_, 1.49 bar N_2_) was used for measuring transmitted light from the reference CeO_2_ employed for energy alignment. The spectra were collected in the 5.5–6.1 keV energy range in step-scan mode with energy-dependent resolution, i.e., 5 eV/point in the pre-edge, 0.5 eV/point in the XANES region, and 1 eV/point in the EXAFS region. The *ex situ* XAS spectra of Pt/*n*CeO_2_ and Pt/UiO-66(Ce) samples were recorded on preactivated pellets (with optimized area and thickness) sealed in LDPE envelopes preventing air contamination (Figure S1a). The pellet activation was the same as described for IR. After activation, the pellets were moved to a N_2_ glovebox without exposing them to air and sealed in LDPE envelopes. Two spectra were measured and averaged. Due to the low Pt content, Pt L_3_-edge was measured in fluorescence mode with a Si drift detector with 13 elements. A metallic Pt foil was parallelly measured in transmission mode on I_2_ for energy alignment. The spectra were recorded in the 11.3–11.8 eV range in step-scan mode with energy-dependent resolution, i.e., 5 eV/point in the pre-edge, 0.5 eV/point in the XANES region, and 1 eV/point in the EXAFS region for a total of 250 points. The integration time was set to 9 s/point for a total of ≈45′/scan. Due to the lower Pt content, 14 scans were averaged for Pt/UiO-66(Ce) spectra while only 2 scans were averaged for the Pt/*n*CeO_2_ sample. It should be point out that as the Pt content was close to the technical limit, to remove Ce L_α,β_ noise contribution, an Al foil of 25 μm thickness was placed in front of the fluorescence detector (Figure S1b). Sample activation procedure was conducted with a microtome reactor cell (Figure S1b). The sample in the form of a pellet was mounted in the cell and heated (5 °C/min) to 110 °C under dry He stream for 2h. After activation, the sample was cooled to RT under He, and the spectra were measured under these conditions (RT, He). The spectra of Pt/*n*CeO_2_ and that of the used catalysts were recorded with the same setup, however, without undergoing the activation procedure. The reference PtO_2_ and PtCl_2_ spectra were recorded in transmission mode from a pellet placed before I_1_.

Spectral analysis (background subtraction and edge-jump normalization) was conducted with the Athena software from the Demeter package.^38^ Linear combination fitting (LCF) procedure was done with the same software in the 5700–5750 eV energy range. Ce(NO_3_)_3_ and UiO-66(Ce) were used as Ce^3+^ and Ce^4+^ references, respectively. The references weight percentage was constrained to the 0–1 interval, while the sum of the weights was not constrained to unity. The sum of the weights was instead used as the indicator of the goodness of fit. FT-EXAFS spectra were extracted in the 3–8.5 Å^–1^ k-range.

## Results and Discussion

### Synthesis and Fundamental Characterization of Pt/UiO-66(Ce)

The structure of the crystalline UiO-66(Ce) material is based on the interaction of hexaoxometallic Ce-based clusters and terephthalate ligands.^33,34^ The preparation of UiO-66(Ce) has been carried out by following previous descriptions in the literature (see the Materials and Methods for details).^33−35^ The PXRD pattern of the achieved solid undoubtedly reveals the crystalline nature of the UiO-66 material (see UiO-66(Ce) in Figure S2), and the FESEM image indicates the formation of homogeneous octahedral crystals with an average particle size distribution of 200–300 nm (see UiO-66(Ce) in Figure S3). N_2_ adsorption characterization shows a type-I isotherm for the synthesized UiO-66(Ce) material (see Figure S4), which is typical of materials with high microporosity. The measured BET surface area, micropore area, and micropore volume are analogous to those reported for well-crystallized UiO-66-type materials (∼1011 m^2^/g, ∼983 m^2^/g, and ∼0.38 cm^3^/g, respectively, see Table S1).^33−35^ Finally, the thermogravimetric profile indicates that the MOF is stable until ∼320 °C under aerobic conditions (see Figure S5).

As stated above, the hexanuclear clusters in Ce-MOFs, particularly in UiO-66(Ce), are populated by hydroxyl groups and structural defects, which are potentially accessible to interact with isolated metal sites.^31,36^ However, in order to efficiently deposit and strongly anchor the single-site Pt^δ+^ species on UiO-66(Ce), we have used a strong electrostatic adsorption strategy by using a simple deposition method with a slightly basic solution, as it has been described for bulk-type metal oxide supports.^39−41^ Otherwise, the repulsive forces between positive metallic species and hydroxyl groups under near-neutral pH conditions could prevent an efficient metal deposition.

The optimal pH to induce a negative charge on the UiO-66(Ce) sample was determined by measuring the zeta potential of the MOF in the 2–10 pH range (see Figure S6). UiO-66(Ce) presents partial negative charges when the pH is >6.5, which will favor the electrostatic interaction with metallic cationic species. Consequently, Pt deposition has been carried out using a bicarbonate solution (a slightly basic solution of pH ∼ 8.3) to ensure the partial deprotonation of the hydroxyl groups (see the Materials and Methods section for details). The resulting material was filtered and washed several times with water to avoid the presence of unbounded Pt species.

The PXRD pattern of the resultant Pt/UiO-66(Ce) shows the characteristic peaks of the UiO-66(Ce) material, discarding any change in the structure during the impregnation process (see Figure S2). Moreover, the characteristic peaks of platinum nanoparticles are absent in the PXRD pattern.^42^ FESEM images clearly indicate that the former octahedral crystals of ∼200−300 nm are maintained after the platinum deposition (see Figure S3). The textural properties of Pt/UiO-66(Ce), measured by N_2_ adsorption, are almost unaltered compared to the values measured for the as-prepared UiO-66(Ce), suggesting that the introduced Pt does not block the micropores of the MOF (see Figure S4 and Table S1). Moreover, some structural defects were generated during the impregnation process, as revealed by the lower weight loss of the impregnated Pt/UiO-66(Ce) compared to the as-synthesized UiO-66 during TGA (see TGA profiles in Figure S5). The treatment with the bicarbonate solution causes the breaking of some Ce–O (cluster–ligand) bonds.^43^ In other words, some of the organic linkers could be shed from the UiO-66 structure, leaving the inorganic cluster more accessible. This fact could also explain the small increment of the surface area when Pt was incorporated on the material. Finally, the ICP analysis confirms a Pt/Ce molar ratio of ∼0.002 on the final Pt/UiO-66(Ce) sample (corresponding to ∼0.07 % wt Pt). At this point, and for comparison purposes, a well-stablished Pt supported on a commercial nanosized CeO_2_, with an analogous Pt/Ce molar ratio of ∼0.004 (corresponding to ∼0.3 % wt Pt), was prepared by the incipient wetness impregnation method (see the Materials and Methods section for details).^37^ The as-obtained material was denoted as Pt/*n*CeO_2_. *n*CeO_2_ employed as the support is based on ∼8.8 nm nanoparticles (average size) and presents a surface area of 98 m^2^/g (see Figure S7).

### Pt–Ce Interaction in Pt/UiO-66(Ce): Unraveling the Electronic State of Ce and Pt

**Figure 1:**
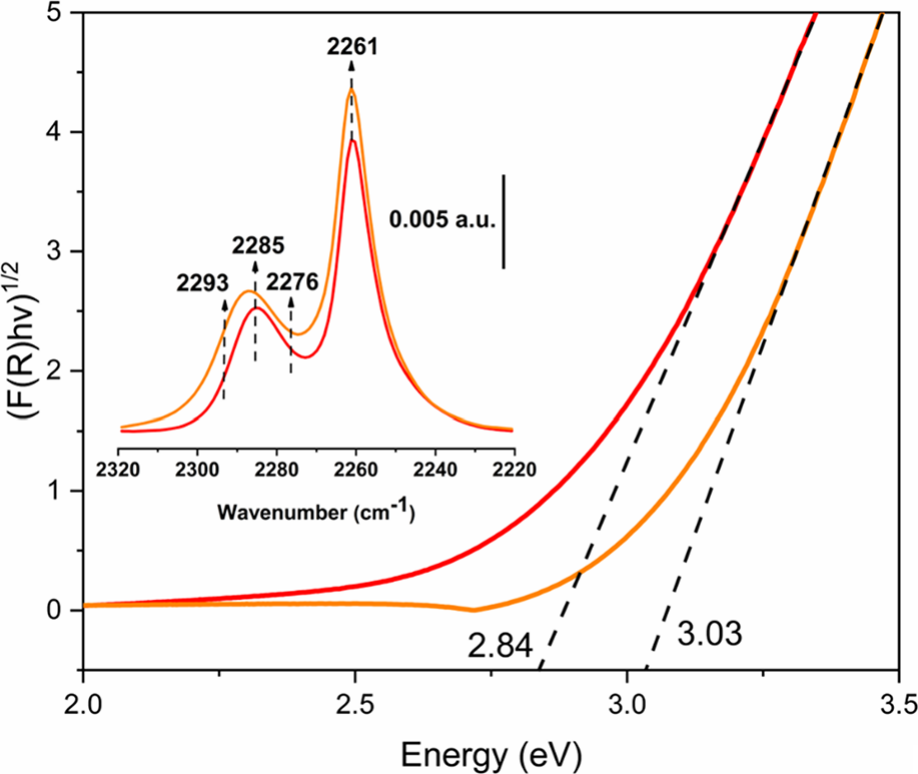
Tauc plots of UiO-66(Ce) (orange line) and Pt/UiO-66(Ce) (red line). Normalized FT-IR spectra of CD_3_CN adsorbed at the maximum coverage on UiO-66(Ce) (orange line) and Pt/UiO-66(Ce) (red line) are reported in the inset.

Fourier transform infrared (FT-IR) spectroscopy has been preliminarily used to evaluate the possible metal–support interactions between Pt^δ+^ and Ce–O^–^ clusters in the final Pt/UiO-66(Ce) material. UiO-66(Ce) and Pt/UiO-66(Ce) samples have been studied by FT-IR spectroscopy. The spectrum of UiO-66(Ce) after being activated at 110 °C under dynamic vacuum (<5 × 10^–4^ mbar) shows the presence of three different bands (Figure S8). The band centered at 3648 cm^–1^ can be assigned to the ν(OH) stretching mode of the (μ_3_-OH)Ce_6_ cluster,^44^ whereas the other two broad bands centered at 3645 and 3637 cm^–1^ can be ascribed to the −OH/–OH_2_ groups of the Ce cluster generated during the structural defect formation.^45^ After Pt introduction, the latter bands disappear, suggesting the potential interaction between Pt and the −OH, −OH_2_ groups, while leaving the (μ_3_-OH)Ce_6_ species unchanged (see Figure S8). The presence of Brønsted and Lewis sites was then probed by CD_3_CN. The effect of CD_3_CN interaction is shown in Figure 1 (inset). Apart from the strong contribution due to the liquid-like CD_3_CN (2261 cm^–1^),^46,47^ the spectra obtained on the two samples are characterized by a broad band at a higher frequency (broader in the case of the UiO-66(Ce) sample). A curve-fitting deconvolution has been carried out in order to estimate the different contributions (see Figure S9). Over the activated UiO-66(Ce) sample, three bands at 2276, 2285, and 2293 cm^–1^ were observed, which are associated to −OH interaction and two different Ce^4+^ Lewis acid sites,^45,48^ respectively. Conversely, in the case of Pt/UiO-66(Ce), the component at higher frequency (2293 cm^–1^) almost disappeared (see Figures 1 and S9), highlighting the consumption of Ce^4+^ sites induced by Pt impregnation.

The functionalization with Pt has a relevant impact, also with respect to the electronic properties, affecting substantially the band gap of the original sample (Figure S10). Pt/UiO-66(Ce) shows a shift to a lower energy of the band gap associated with the electronic transfer from the 2p valence band of O^2–^ to the 4f level in the conduction band of Ce^4+^.^49^ Interestingly, this decrease in the band gap when comparing the UiO-66(Ce) and Pt/UiO-66(Ce) materials (from 3.03 to 2.84 eV, respectively), could be associated to the increment of Ce^3+^ species (see Figure 1),^50^ suggesting an increment of oxygen vacancies after Pt impregnation.

To determine the Ce and Pt oxidation states, we collected XPS spectra at Ce(3d) and Pt(4f) binding energies (Figure S11). The Ce(3d) XPS spectrum of the as-prepared Pt/UiO-66(Ce) sample shows a slightly higher Ce^3+^ content (∼6%) compared to Pt/*n*CeO_2_ (∼3%) (Figure S11). However, as we previously reported, Ce^3+^ quantification is not straightforward due to the intrinsic reduction during the measurement.^50^ The Pt(4f) region on Pt/*n*CeO_2_ showed two peaks at 72.8 and 76.1 eV, ascribable to the Pt^2+^ 4f_7/2_ and 4f_5/2_ bands (Figure S11d). On the contrary, the Pt content in Pt/UiO-66(Ce) was too low to observe the Pt(4f) peaks (Figure S11e).

**Figure 2:**
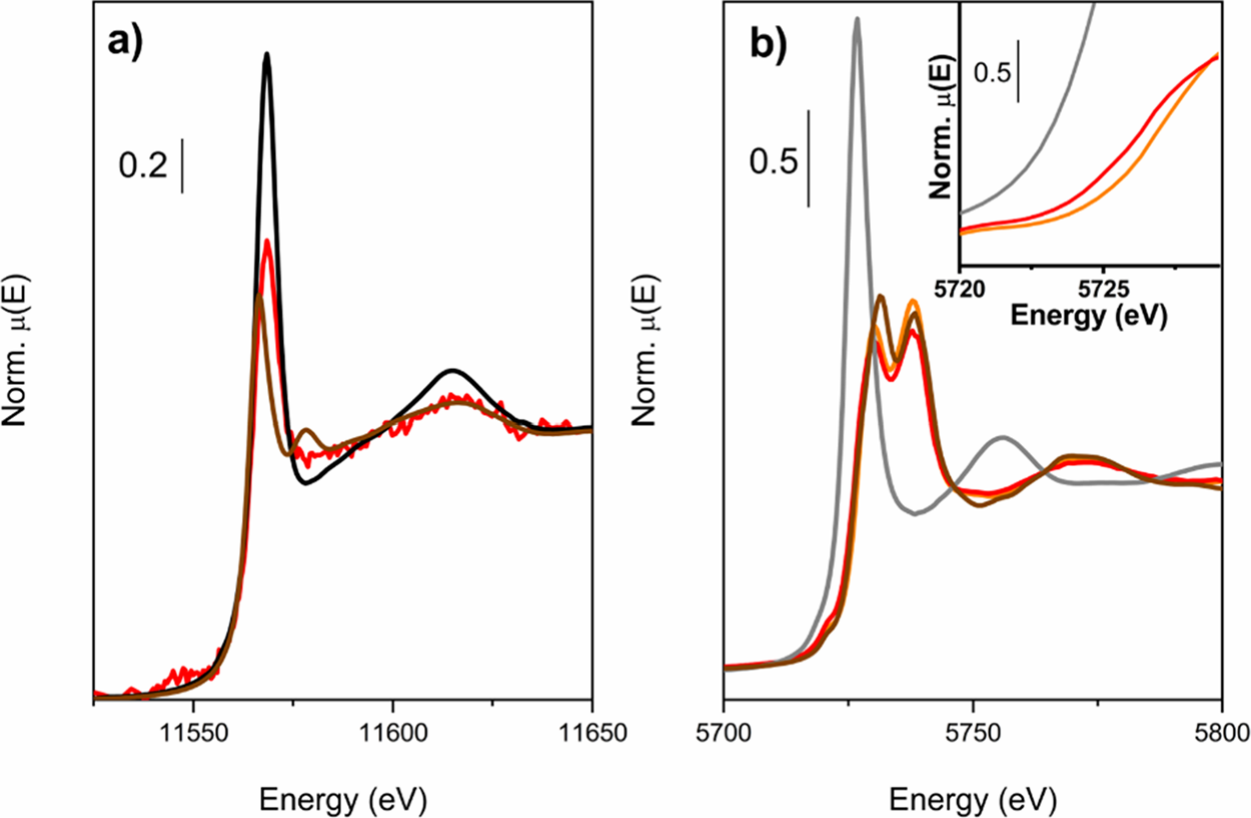
(a) Pt L_3_-edge XANES experimental spectra of Pt/UiO-66(Ce) (red line), PtO_2_ (black line), and PtCl_2_ (brown line). (b) Ce L_3_-edge XANES experimental spectra with the white-line detail in the inset. Pt/UiO-66(Ce) and reference CeO_2_, Ce(NO_3_)_3_, and UiO-66(Ce) spectra are reported in red, brown, gray, and orange lines, respectively.

To further investigate the oxidation states, Ce and Pt L_3_-edge XANES spectra were measured (see the Materials and Methods section for details). In situ Pt L_3_-edge spectra were collected at RT on the activated catalyst (Figure 2a). Even though the low Pt content led to noisy spectra, the white-line intensity observed between Pt^4+^ and Pt^2+^ references indicates a Pt^δ+^ oxidation state (2+ < δ < 4+).^32,51^ Interestingly, Pt/*n*CeO_2_ also presented Pt L_3_-edge XANES spectra comparable with the Pt/UiO-66(Ce) one (Figure S12). However, the higher white-line intensity detected on the Pt/*n*CeO_2_ spectrum suggests an average Pt oxidation state higher than Pt^δ+^. It is noteworthy that in the Pt/UiO-66(Ce) sample, a weak component was observed in the spectral pre-edge around 11,540 eV, which is ascribable to the W L_2_-edge signal.^52^ Indeed, W is one of the components of the microtome heating element, and considering the low Pt content/long measurement time, it is reasonable to observe its weak signal. Differently, *ex situ* Ce L_3_-edge XANES spectra were measured at RT on previously activated and sealed pellets (Figure 2b). The spectrum collected for the UiO-66(Ce) sample is comparable to the one obtained for the CeO_2_ reference. Contrarily, Pt/UiO-66(Ce) presented a shoulder in the rising-edge region ascribable to Ce^3+^, where Ce(NO_3_)_3_ was employed as the reference (Figure 2b inset). A linear combination fit (LCF) analysis revealed that the Pt/UiO-66(Ce) spectrum could be described by a combination of 8% Ce(NO_3_)_3_ and 92% UiO-66(Ce) (Figure S13), highlighting an increase of Ce^3+^ content after the Pt impregnation procedure, as suggested by the UV–vis results.

**Scheme 1:**
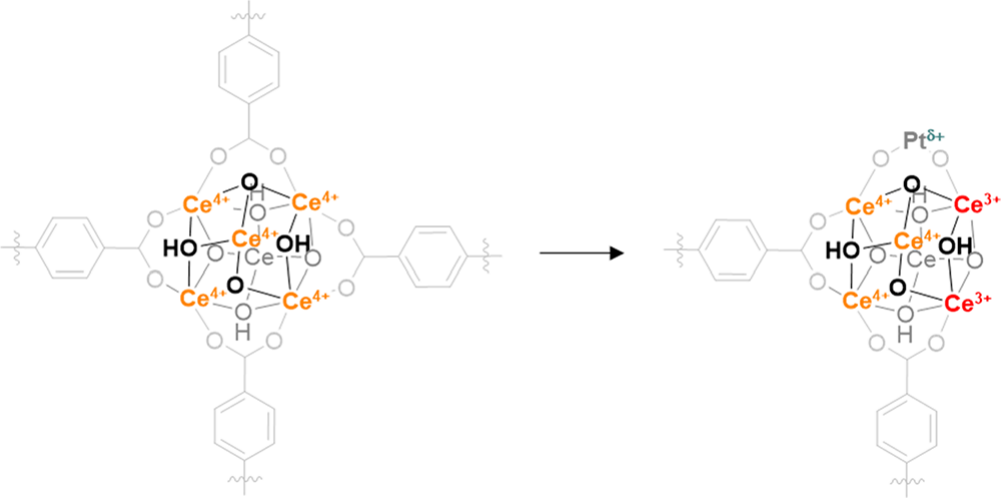
Active Sites Generated during Pt Anchoring on the Ce Cluster

By considering the amount of loaded Pt and the number of missing linkers after Pt anchoring (2%, determined by TGA), we could estimate that only ∼10% of the CeO_*x*_ clusters presenting missing linkers have grafted Pt. This indicates that the grafted Pt and missing linkers coexist within the same catalyst, explaining why Ce^3+^ was observed in the Pt/UiO-66(Ce) catalyst (see Scheme 1). With this configuration, the proximity between Ce^3+^ and Pt^δ+^ could be adequate to activate the oxygen molecules and carbon monoxide molecule,^53^ respectively, and, consequently, the Pt/UiO-66(Ce) material could be an ideal catalyst for the CO oxidation reaction, as will be presented later.

**Figure 3:**
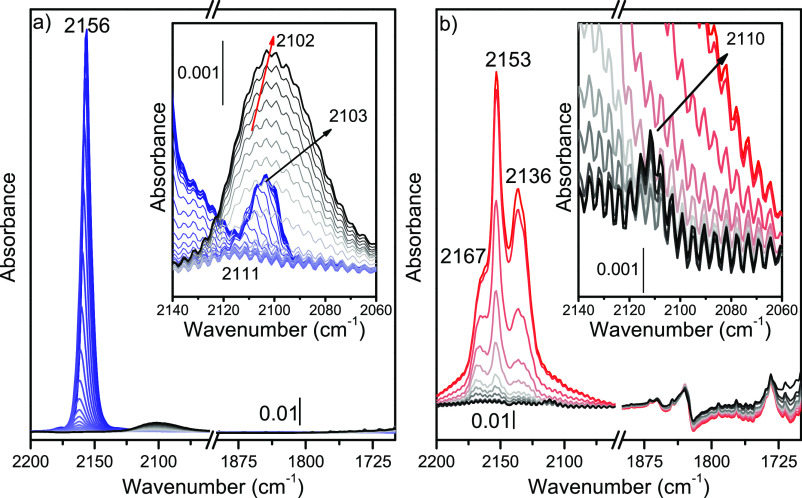
Difference of FT-IR spectra of heating CO (from LNT to RT): (a) Pt/*n*CeO_2_ and (b) Pt/UiO-66(Ce). Detail of CO–Pt vibration is reported in the inset. Temperature increases from blue/red color to black color.

To confirm and investigate the nature of the Pt species, CO adsorption was studied by FT-IR spectroscopy. Indeed, CO is a well-known probe molecule able to provide information on the Pt nature such as oxidation state and coordination. As reference samples, we first measured CO adsorption at nominal 100 K on *n*CeO_2_ and Pt/*n*CeO_2_ (Figure S14). The spectral profile of the two samples was very similar, both presenting at low partial pressures a single broad band at 2157 and 2162 cm^–1^, respectively, shifting to lower wavenumbers while pressure rises. While the band shift is a well-known phenomenon related to the coverage effect,^54^ the hypsochromic shift of the initial band in Pt/*n*CeO_2_ indicates a different nature of the catalyst surface. Indeed, the band position is related to CO–Ce^4+^ interaction,^55,56^ and its formation at higher wavenumbers indicates a higher δ-donation (from CO to Ce^4+^). This highlights the presence of Ce^4+^ CUS sites likely formed after the Pt grafting procedure. Moreover, both *n*CeO_2_ and Pt/*n*CeO_2_ presented a weak band at 2103 cm^–1^ ascribed to ^13^CO–Ce^4+^ contribution (^13^CO–Ce^4+^ frequency is 0.9777 of ^12^CO–Ce^4+^).^57^ However, a broad band at 2111 cm^–1^, which could be related to CO–Pt interaction, was only detected on the Pt/*n*CeO_2_ sample. Indeed, the band position and its weak intensity are in line with the literature results and the low Pt concentration.^58,59^ To clarify this band assignment, crucial for disclosing the Pt nature, we monitored the CO spectra evolution while heating the sample from 100 K to RT (Figure 3a). Indeed, CO at low temperature is adsorbed on both Ce^4+^ and Pt, while it is selectively adsorbed on Pt sites at RT. During heating, CO was quickly desorbed from Ce^4+^ sites. Moreover, the band at 2103 cm^–1^ decreased similarly in intensity, confirming its relation to ^13^CO vibration. Contrarily, the band at 2111 cm^–1^ showed an initial higher stability, in line with the strong CO–Pt interaction while it suddenly increased and shifted to 2102 cm^–1^. The bathochromic shift suggests that Pt is partially reduced during heating.^60^ At the same time, in the 1800–800 cm^–1^ region, we observed the formation of the carbonate characteristic vibrations, indicating that Pt reduction occurs through CO-to-CO_2_ transformation (Figure S15). It is noteworthy that any contribution was observed in the multibonded carbonyl region (1900–1700 cm^–1^), suggesting the presence of isolated Pt sites.^61^ Hence, by combining XPS and XANES results, we can associate the 2111 cm^–1^ vibration to CO interacting with the isolated cationic Pt sites.^62^ Taking this assignment as the reference for Pt single sites over ceria, the same experiments were repeated on UiO-66(Ce) and Pt/UiO-66(Ce) samples. After CO adsorption, UiO-66(Ce) and Pt/UiO-66(Ce) showed similar FT-IR spectra (see Figure S16), presenting two main bands at 2153 and 2136 cm^–1^, corresponding to CO interaction with the hydroxyl groups and physisorbed CO, respectively.^63,64^ However, an extra band at 2167 cm^–1^ was present only for the Pt/UiO-66(Ce) sample (Figure S16b). The shift to higher wavenumber of this band with respect to noninteracting CO (≈2141 cm^–1^) indicates an important σ-donation contribution, which can be ascribed to the Ce^4+^ coordinative unsaturated sites (CUS) generated during the missing linker process.^65,66^ The OH region of both samples was consumed with the increase of CO partial pressure, while a broad band arose at about 3565 cm^–1^, confirming the CO interaction with the hydroxyl groups (Figure S16 inset). Following the previous experiment on Pt/*n*CeO_2_, CO–Pt contribution is expected to be convoluted with the ^13^CO vibration around 2110–2103 cm^–1^. Nevertheless, we could not appreciate these bands in Pt/UiO-66(Ce) likely due to the important contribution of the physisorbed CO signal. To identify the CO–Pt interaction over Pt/UiO-66(Ce), we then repeated the same heating experiment performed on Pt/*n*CeO_2_ (Figure 3b). As observed on the reference sample, during heating, CO is quickly desorbed from Ce^4+^ and OH sites. Contrarily, we clearly noticed a band at ∼2110 cm^–1^ that persisted up to RT, confirming the presence of the CO–Pt interaction. Moreover, following Pt/*n*CeO_2_ assignment and Pt L_3_-edge XANES results, the 2110 cm^–1^ band was associated to CO interacting with the isolated cationic Pt^δ+^ sites. It is noteworthy as these sites are not reduced during heating under CO, while they are reduced in the Pt/*n*CeO_2_ case, suggesting a remarkably higher stability of Pt in Pt/UiO-66(Ce) with respect to Pt/*n*CeO_2_.

### CO Oxidation Reaction Using Pt/UiO-66(Ce) and Pt/*n*CeO_2_: Important Role of Pt–Ce Interaction

**Figure 4:**
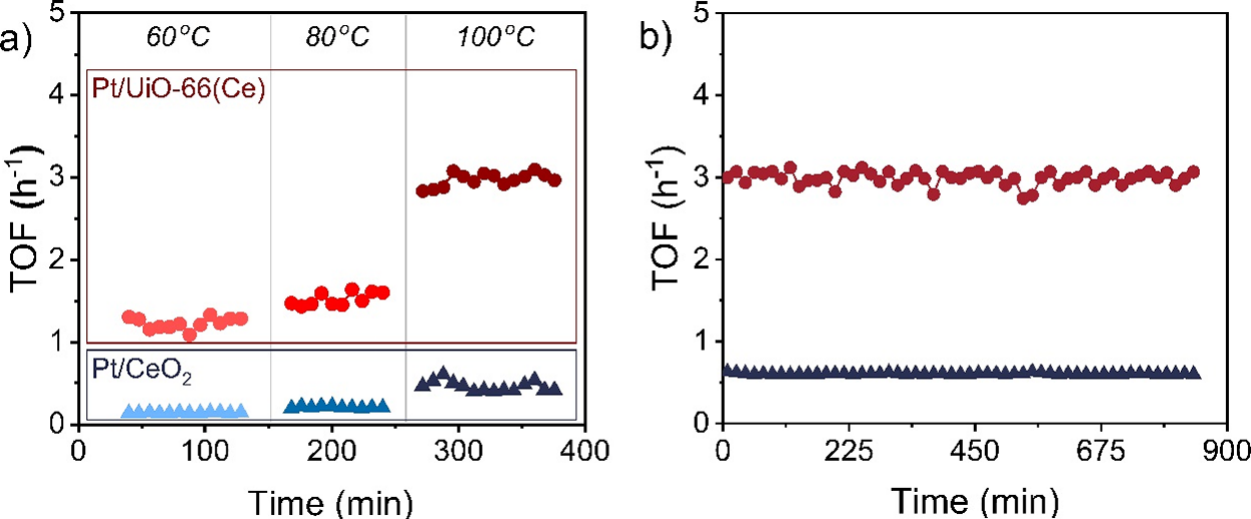
(a) Calculated TOFs for CO oxidation using Pt/UiO-66(Ce) (circles) and Pt/*n*CeO_2_ materials (triangles) at different reaction temperatures and the following reaction conditions (GHSV = 20,000 mL/g_cat_·h, 0.6% CO, 9.6% O_2_). (b) Long time-on-stream (TOS) curves of Pt/UiO-66(Ce) (circles) and Pt/*n*CeO_2_ (triangles) at 100 °C and the following reaction conditions (GHSV = 20,000 mL/g_cat_·h, 0.6% CO, 9.6% O_2_).

Considering the differences between Pt/UiO-66(Ce) and Pt/*n*CeO_2_ in redox properties, supporting the reducibility and stability of Pt cationic single sites under CO conditions, both materials have been initially tested as catalysts for the low-temperature CO oxidation reaction in a continuous-flow fixed-bed reactor with a feed containing 0.6% CO and 9.6% O_2_ and a selected GHSV of 20,000 mL/g_cat_·h. The obtained CO conversion values for both materials under these low-temperature conditions were very low (below 1%, see Table S2), as also reported in the literature for other single-atom catalysts.^37^ However, a clear increase of the TOF value (calculated as moles of CO_2_ per mole of Pt in an hour) is observed for the Pt/UiO-66(Ce) catalyst within the 60–100 °C range, and, compared to Pt/*n*CeO_2_, a ∼sixfold TOF increment is achieved when using the Pt/UiO-66(Ce) catalyst at these reaction temperatures (see Figure 4 and Table S2). As summarized in Table S3, the Pt/UiO-66(Ce) single-atom catalyst presents improved TOF values for CO oxidation compared to other representative isolated cationic metal-supported MOF catalysts reported in the literature at these low-temperature reaction regimes.^30,36,67^

To unavoidably assess the catalytic differences between the Pt/UiO-66(Ce) and Pt/*n*CeO_2_ catalysts, the CO oxidation reaction has also been evaluated at lower GHSV (10,000 mL/g_cat_·h; feed: 25 mL/min, 0.6% CO; 9.6% O_2_) and at similar GHSV but using a more diluted CO feed (22,000 mL/g_cat_·h; feed: 55 mL/min, 0.08% CO; 4.0% O_2_). As summarized in Table S2 and Figure S17, the sixfold TOF increase when using Pt/UiO-66(Ce) as the catalyst over Pt/*n*CeO_2_ is still observable in all cases.

The calculated activation energies for Pt/UiO-66(Ce) and Pt/*n*CeO_2_ were 22.0 and 28.1 kJ/mol, respectively (see Figure S18). These values are similar to those obtained in the literature for the well-dispersed Pt single sites on ceria.^68^ The ∼6.1 kJ/mol decrease on the apparent activation energy underlines the fact that Pt^δ+^ supported on the Ce-based MOF is an active catalyst for the low-temperature CO oxidation.

Typically, the CO oxidation process could be affected by many factors, such as the catalyst surface area, catalyst reducibility at low temperature, or the chemical state of the surface element, among others.^23^ Consequently, the highest observed activity for the Pt/UiO-66(Ce) catalyst compared to Pt/*n*CeO_2_ could not be unambiguously assigned to an individual factor. However, the elevated surface area of the MOF support, the presence of a greater amount of Ce^3+^ species, and, particularly, the strongest interaction between Pt^δ+^ and Ce–O^–^, demonstrated by different spectroscopic techniques, would play determinant roles to explain the improved catalytic performance of Pt/UiO-66(Ce) for the low-temperature CO oxidation reaction.

As can be deduced from the PXRD pattern of the recovered Pt/UiO-66(Ce) after 2 h on the CO oxidation stream at 100 °C (see Figure S19), the sample mostly retains its crystalline nature, and no evidence of Pt aggregation was found, while FE-SEM images show the maintenance of the crystal morphology (see Figure S20). Although a decrease of the surface area is observed after the catalytic evaluations (see Figure S21 and Table S4), the catalytic performance is not severely affected since the activity remains almost unaltered. Noticeably, the Pt L_3_-edge XANES spectra collected after Pt/UiO-66(Ce) and Pt/*n*CeO_2_ catalytic tests did not show relevant differences with the original catalysts (Figure S22), indicating a good stability of the Pt species. Finally, a long-term stability test at 100 °C for the CO oxidation reaction also demonstrated that the activity was preserved after 14 h (see Figure 4b). All these findings illustrate the stability of the Pt species strongly supported on the Ce cluster for the low-temperature CO oxidation reaction. Based on these excellent preliminary results, preparing Ce-MOF structures with improved crystalline stability to design highly active and stable SAC catalysts with unique catalytic performances is currently a subject of great interest within our groups.

## Conclusions

Herein, we reported the synthesis of a single Pt site catalyst supported on the UiO-66(Ce) material. Zeta-potential analysis disclosed that the hydroxyl groups presented on the CeO_*x*_ MOF clusters could be deprotonated under basic pH values. As was demonstrated by FT-IR spectroscopy, these −OH/–OH_2_ groups generated during the missing-linker process are consumed after Pt deposition, suggesting a Pt–MOF interaction. Moreover, during this process, an increment of oxygen vacancies was deduced by the decrease on the band gap and by Ce L_3_-edge XANES spectroscopy, where the contribution of Ce^3+^ species increased after the Pt incorporation. CO adsorption, followed by IR, and Pt L_3_-edge XANES spectroscopy, corroborate the presence of cationic Pt^δ+^ species supported on the UiO-66(Ce) material (2+ < δ < 4+). To demonstrate the unique redox properties of the Pt/UiO-66(Ce) material, we have synthesized and fully characterized a well-known Pt single-site catalyst, denoted as Pt/*n*CeO_2_. In the last case, the higher white-line intensity detected on the Pt L_3_-edge XANES spectrum suggests an average Pt oxidation state higher than that observed for Pt/UiO-66(Ce). Based on the promising redox characteristics of Pt/UiO-66(Ce), this material was studied as the catalyst for the low-temperature CO oxidation reaction. Normalized activities per Pt site showed an ∼six-fold catalytic activity increment compared to the Pt/*n*CeO_2_ catalyst. Finally, Pt L_3_-edge XANES analyses reveal that the Pt nature is preserved after the catalytic evaluation.

## Supporting Information Available

Characterization of the materials including XAS experimental setup, PXRD patterns, FESEM images, N_2_ adsorption isotherms, TGA, zeta-potential measurements, FTIR spectra, UV–vis spectra, XPS spectra, and XANES spectra and catalytic testing including catalytic evaluation, Arrhenius plots, comparison of the catalytic performance with other single-site MOF-based catalysts, and characterization of the material after catalysis (PDF)
