# Supplementary material for: Synergic Effect of Isolated Ce3+ and Ptδ+ Species in UiO-66(Ce) for Heterogeneous Catalysis
Source: ACS Catal. 2023 Jun 26;13(13):9171–80. doi: 10.1021/acscatal.3c00502 (PMC10334465; doi:10.1021/acscatal.3c00502)
Supplement: Supplementary file 1 — cs3c00502_si_001.pdf [file cs3c00502_si_001.pdf]

Supplementary Information

Synergic effect of isolated Ce^{3+} and $Pt^{\delta+}$ species in UiO-66(Ce) for heterogeneous catalysis

Sergio Rojas-Buzo[1], Benjamin Bohigues[2], Davide Salusso[1,3], Avelino Corma[2], Manuel Moliner[2]* and Silvia Bordiga[1]*

[1] Department of Chemistry and NIS Centre, University of Turin, Via Giuria 7, 10125 Turin, Italy

[2] Instituto de Tecnología Química, Universitat Politècnica de València-Consejo Superior de Investigaciones Científicas, Avenida de los Naranjos s/n, 46022 València, Spain

[3] European Synchrotron Radiation Facility, CS 40220, 38043 Grenoble Cedex 9, France

*Corresponding authors: E-mail address: mmoliner@itq.upv.es; silvia.bordiga@unito.it

Table of contents

1. Sample Characterization 3

1.1. XAS experimental set-up 3

1.2. Powder X-ray diffraction (PXRD) patterns 4

1.3. Field Emission Electron Microscopy (FESEM) images 5

1.4. N_2 adsorption isotherms 6

1.5. Thermogravimetric analysis (TGA) 7

1.6. Zeta potential measurements 8

1.7. *n*CeO_2 characterization 9

1.8. FTIR spectra of the activated MOFs 10

1.9. CD_3CN-FTIR spectra 11

1.10. UV-Vis spectra 12

1.11. XPS spectra 13

1.12. XANES spectra 14

1.13. CO-FTIR spectra 16

2. CO oxidation reaction 19

2.1. Catalytic evaluation of Pt/UiO-66(Ce) and Pt/*n*CeO_2 at different conditions 19

2.2. Comparison of the catalytic performance for the Pt/UiO-66(Ce) SAC (this work) with other state-of-the-art single-atom MOF-based representative works. 21

2.3. Arrhenius plots 22

2.4. Pt/UiO-66(Ce) characterization after catalysis 23

3. References 27

1. Sample Characterization

1.1. XAS experimental set-up

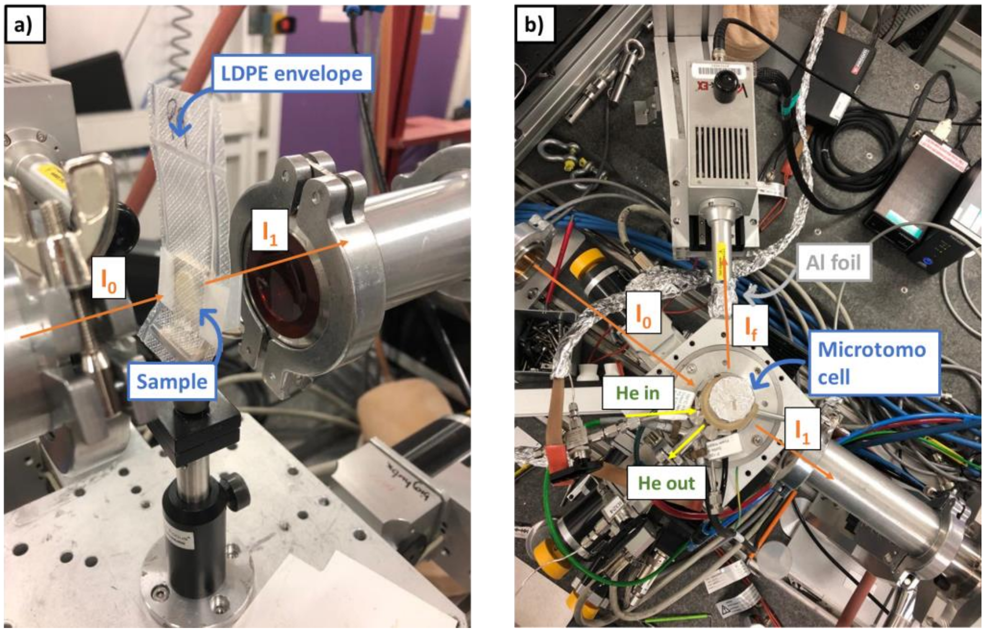

Figure S1. a) Ce L_3-edge and b) Pt L_3-edge experimental set-up.

1.2. Powder X-ray diffraction (PXRD) patterns

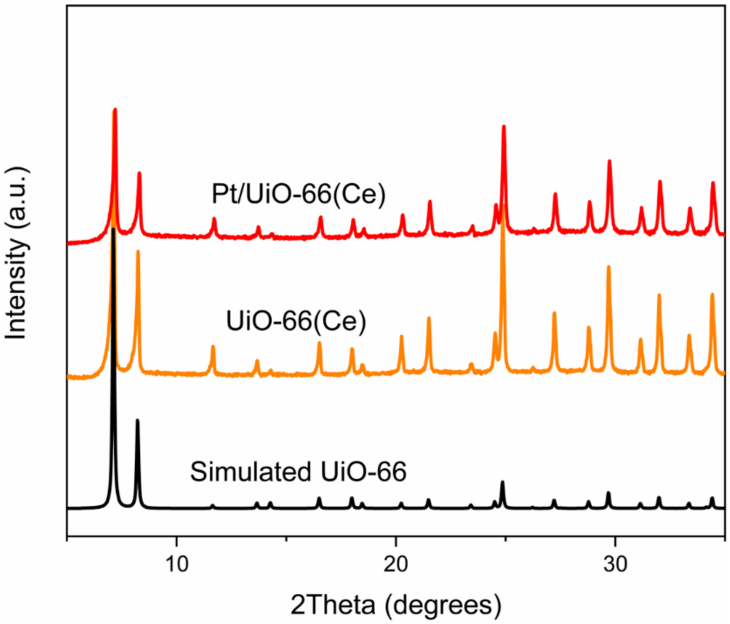

Figure S2. PXRD patterns of the simulated UiO-66(Ce) (black line), as-synthesized UiO-66(Ce) (orange line) and Pt/UiO-66(Ce) (red line).

1.3. Field Emission Electron Microscopy (FESEM) images

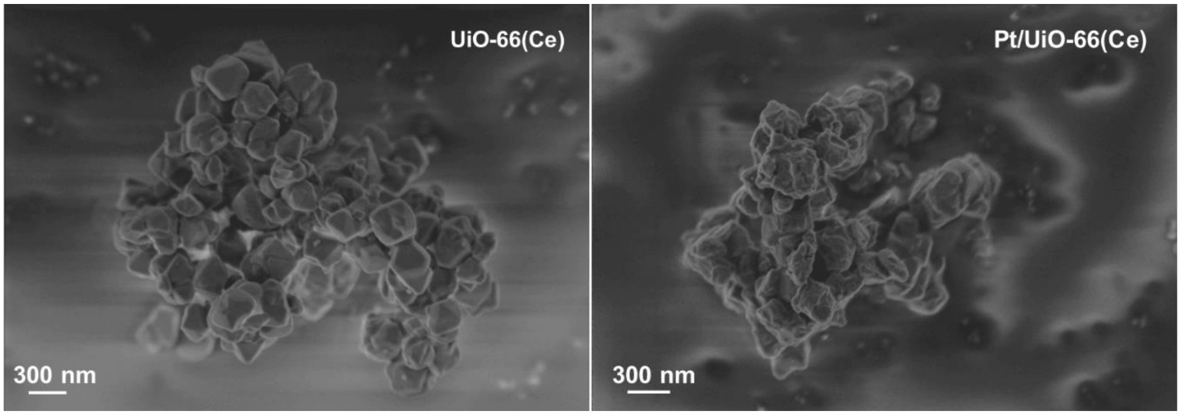

Figure S3. FESEM images of the UiO-66(Ce) (left) and Pt/UiO-66(Ce) (right).

1.4. N_2 adsorption isotherms

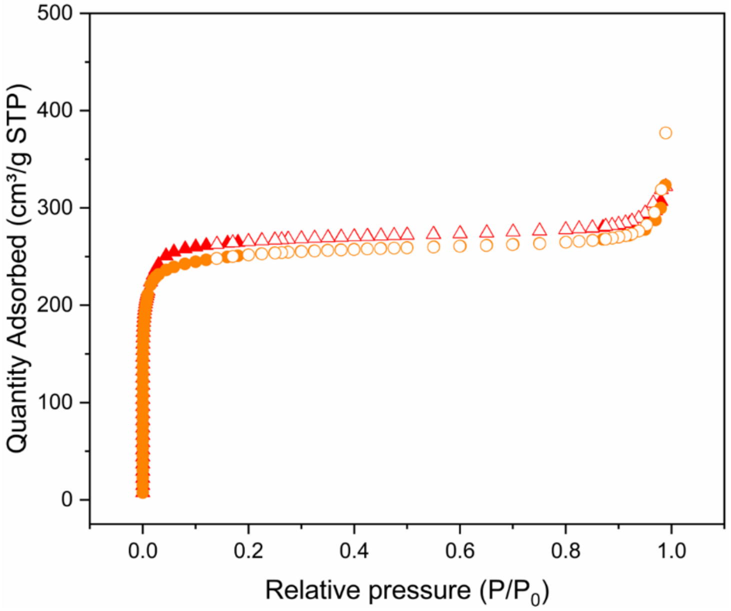

Figure S4. N_2 adsorption-desorption isotherms of UiO-66(Ce) (orange circles) and Pt/UiO-66(Ce) (red triangles).

Table S1. N_2 adsorption isotherm report of before and after MOF impregnation, respectively.

Sample	BET surf. area (m^2/g)	Micro. area (m^2/g)	Micro. volume (cm^3/g)
UiO-66(Ce)	1011	983	0.38
Pt/UiO-66(Ce)	1071	1042	0.40

1.5. Thermogravimetric analysis (TGA)

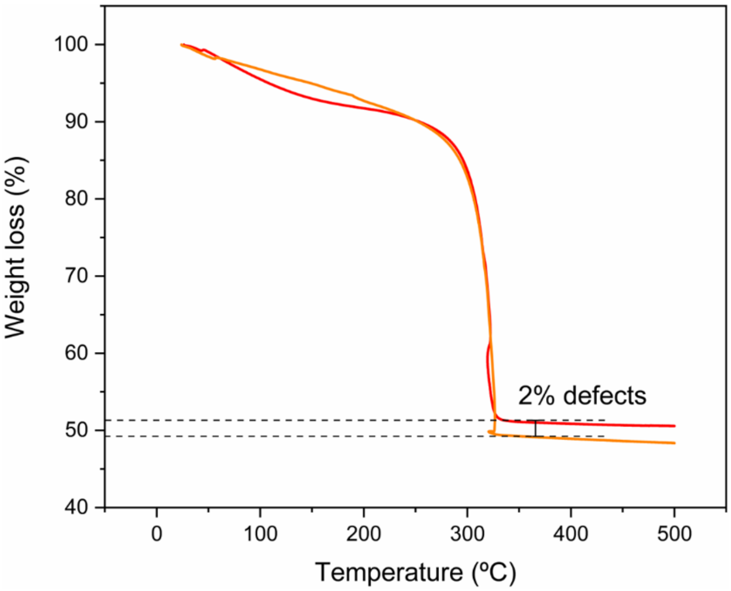

Figure S5. TGA profiles (under aerobic conditions) of the as-synthesized UiO-66(Ce) (orange line) and Pt/UiO-66(Ce) (red line).

1.6. Zeta potential measurements

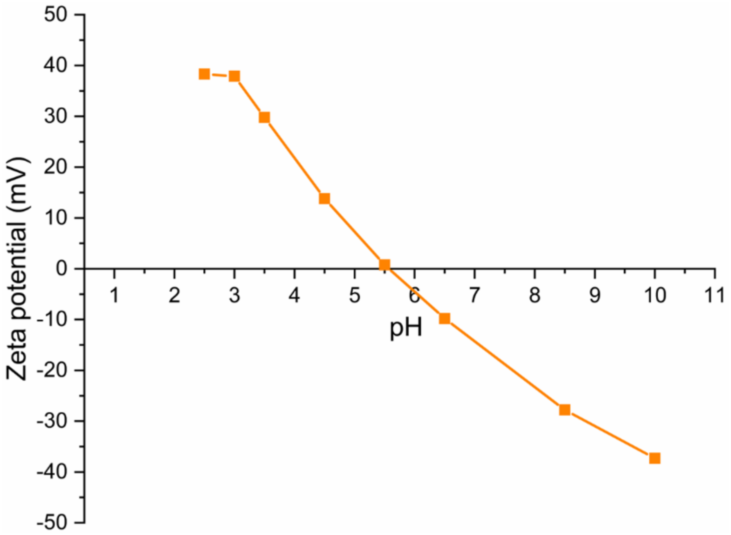

Figure S6. The zeta potential of UiO-66(Ce) as a function of pH.

1.7. *n*CeO_2 characterization

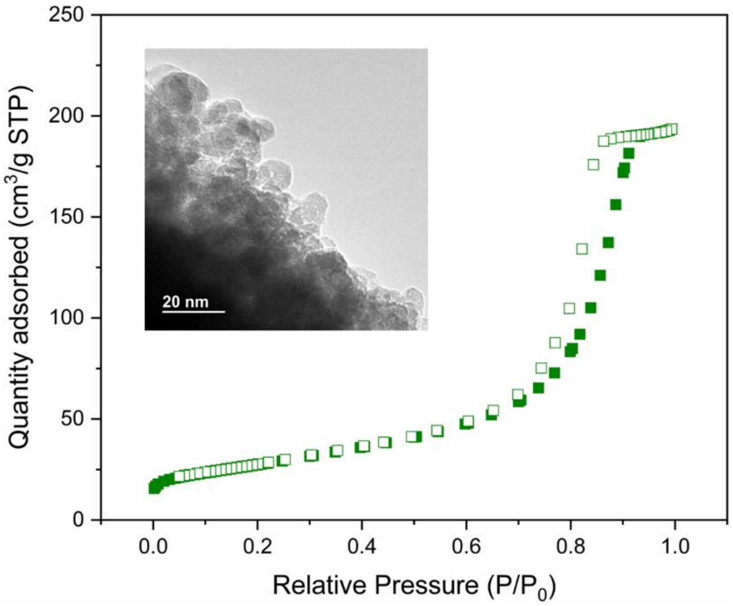

Figure S7. N_2 adsorption-desorption isotherms of *n*CeO_2. HR-TEM image of the *n*CeO_2 is reported in the inset.

1.8. FTIR spectra of the activated MOFs

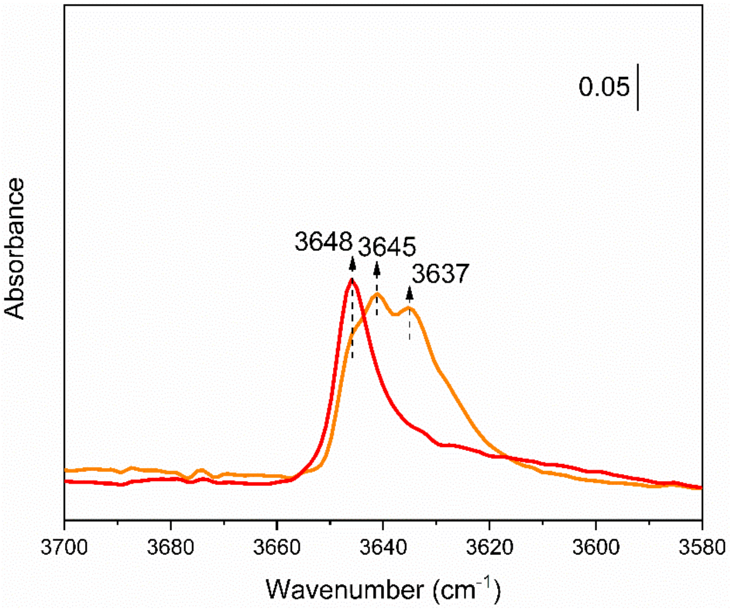

Figure S8. FT-IR spectra of the activated UiO-66(Ce) (orange line) and Pt/UiO-66(Ce) (red line) focused on the OH region.

1.9. CD_3CN-FTIR spectra

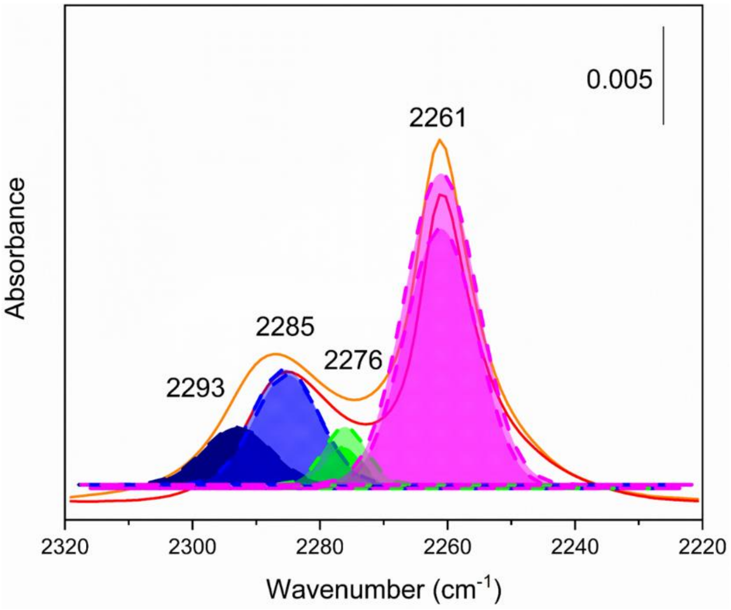

Figure S9. FT-IR fitting of CD_3CN adsorbed at maximum coverage on UiO-66(Ce) (orange line) and Pt/UiO-66(Ce) (red line).

1.10. UV-Vis spectra

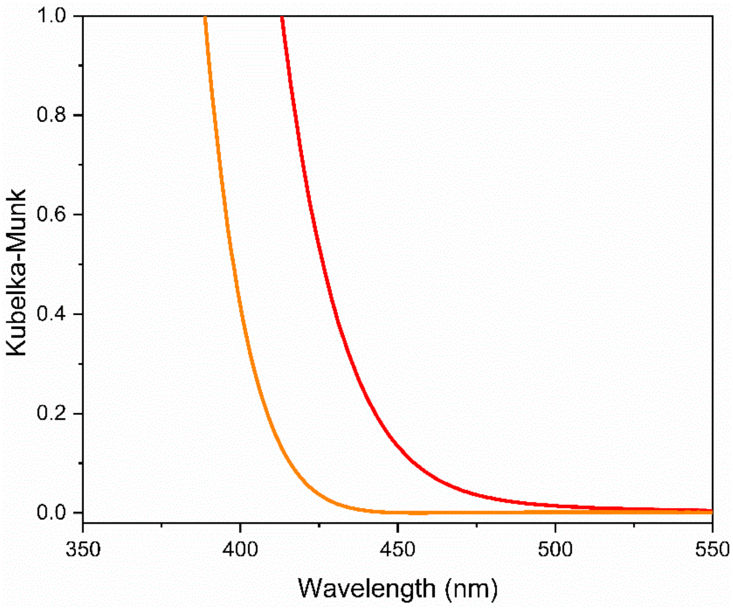

Figure S10. DRS UV-Vis spectra of the UiO-66(Ce) (orange line) and Pt/UiO-66(Ce) (red line) in Kubelka-Munk units.

1.11. XPS spectra

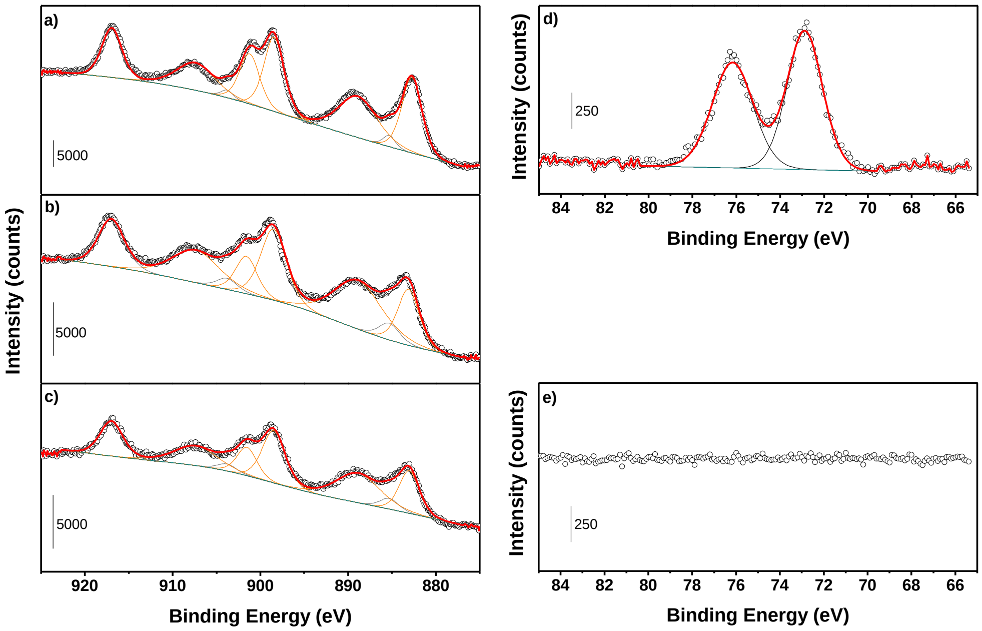

Figure S11. a,b,c) Ce(3d) and d,e) Pf(4f) XPS experimental spectra (black line) and best fit (red line) for a,d) Pt/*n*CeO_2 and b) UiO-66(Ce) and c,e) Pt/UiO-66(Ce) samples. Ce^{4+} and Ce^{3+} components are showed in orange and grey lines, respectively. Background is reported with dark cyan line.

1.12. XANES spectra

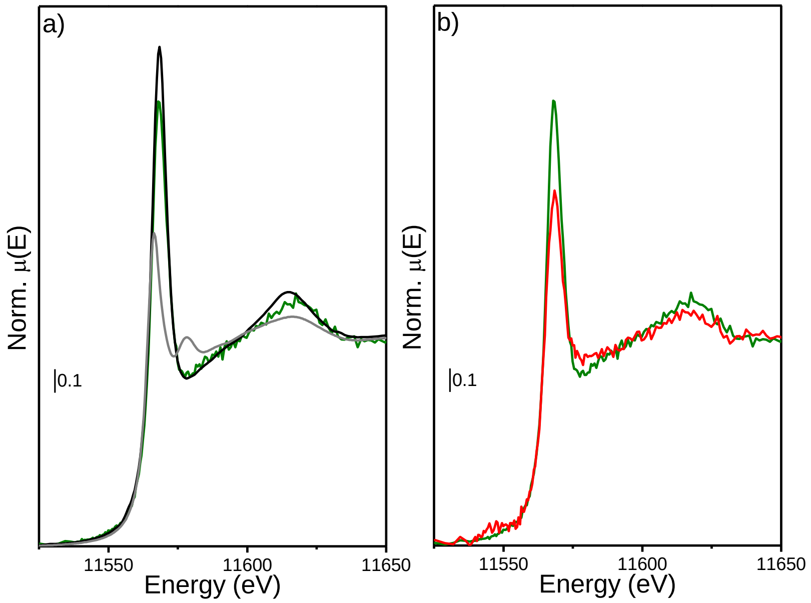

Figure S12. Pt L_3-edge XANES experimental spectra of a) Pt/*n*CeO_2 (green line), $PtCl_2$ (grey line) and PtO_2 (black line) and b) comparison between Pt/*n*CeO_2 (green line) and Pt/UiO-66(Ce) (red line).

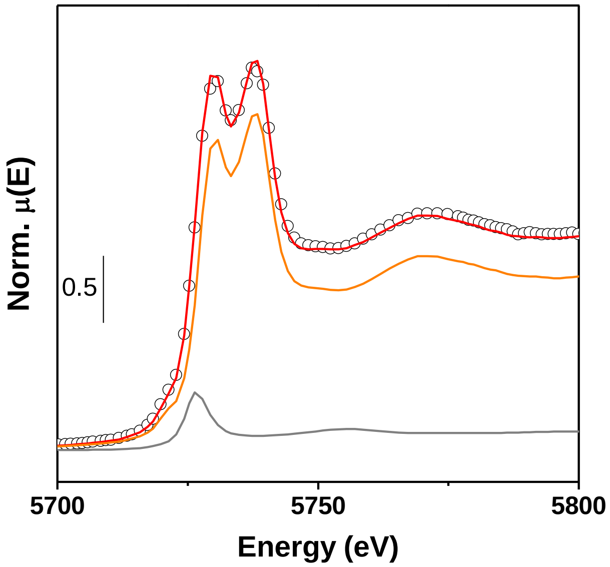

Figure S13. Pt/UiO-66(Ce) Ce L_3-edge experimental XANES spectra (black circles) and LCF best fit (red line). UiO-66(Ce) and Ce(III)$(NO_3)_3$ reference spectra are reported in orange and grey line, respectively.

1.13. CO-FTIR spectra

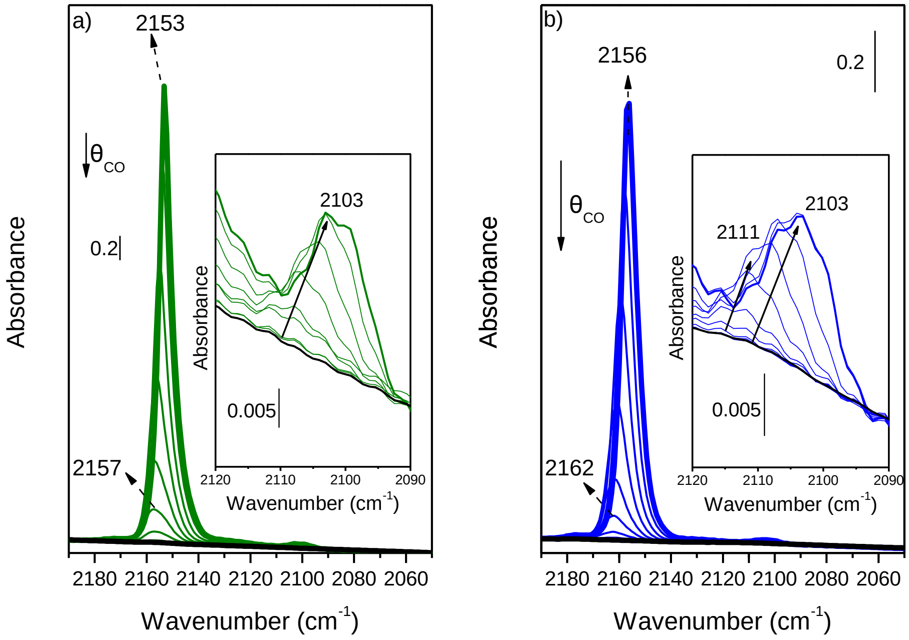

Figure S14. FT-IR spectra of CO desorption at LNT on a) *n*CeO_2 and b) Pt/*n*CeO_2.

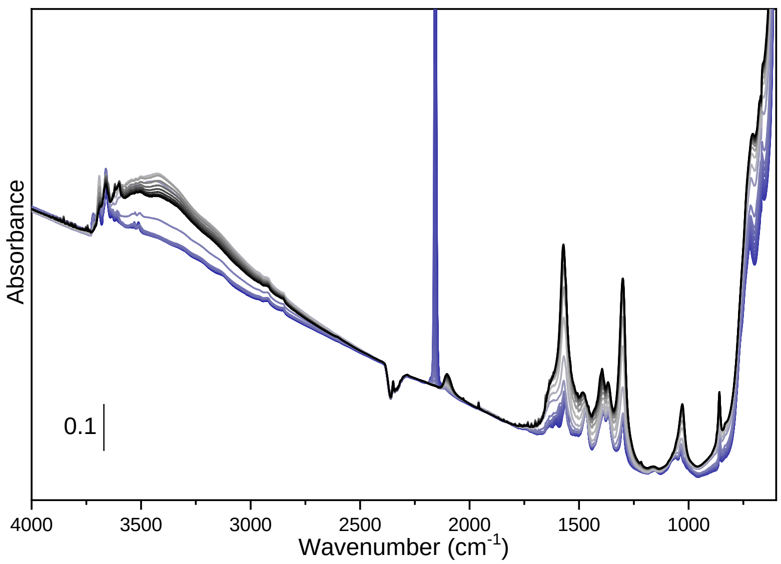

Figure S15. Full range FT-IR spectra of CO (0.085 mbar) obtained for the Pt/*n*CeO_2 during the transition from LNT to RT (from blue to black).

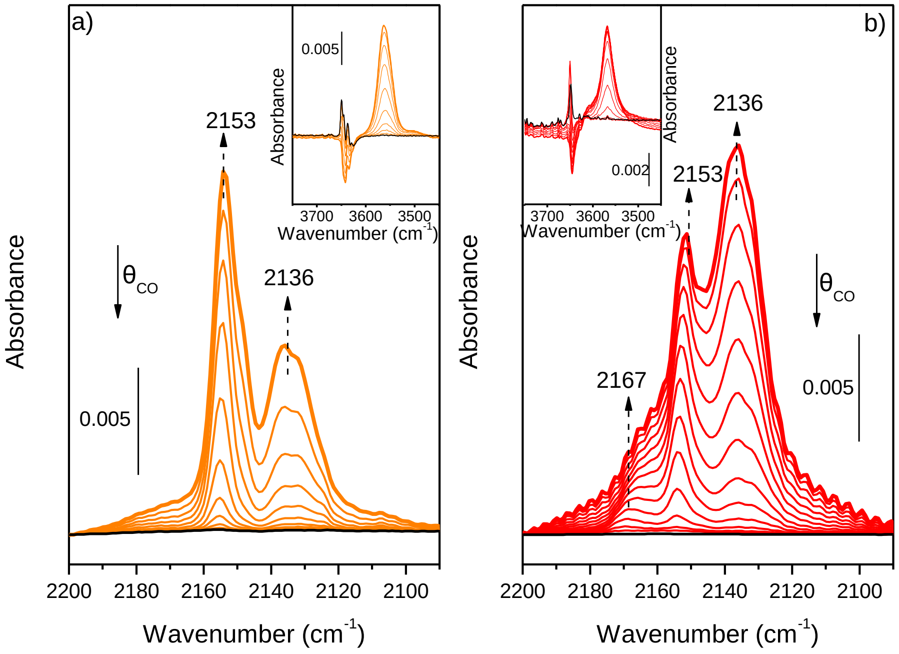

Figure S16. Difference FT-IR spectra of CO desorption at LNT on the a) UiO-66(Ce) and b) Pt-UiO-66(Ce). Details of OH regions are reported in the insets.

2. CO oxidation reaction

2.1. Catalytic evaluation of Pt/UiO-66(Ce) and Pt/*n*CeO_2 at different conditions.

Table S2. Conversions and TOFs of both materials with different feeds at 100°C.

**Catalysts**	**GHSV (mL/g_{cat}·h)**	**Feed**	**X_{CO} (%)**	**TOF (h^{-1})**
Pt/UiO-66(Ce)	20000	0.3 mL CO/min (50 mL/min - 0.6% CO/9.6% O_2)	0.31	3.03
	10000	0.15 mL CO/min (25 mL/min - 0.6% CO/9.6% O_2)	0.64	3.09
	22000	0.045 mL CO/min (55 mL/min – 0.08% CO/4% O_2)	2.62	3.76
Pt/*n*CeO_2	20000	0.3 mL CO/min (50 mL/min - 0.6% CO/9.6% O_2)	0.18	0.50
	10000	0.15 mL CO/min (25 mL/min - 0.6% CO/9.6% O_2)	0.41	0.56
	22000	0.045 mL CO/min (55 mL/min – 0.08% CO/4% O_2)	1.56	0.64

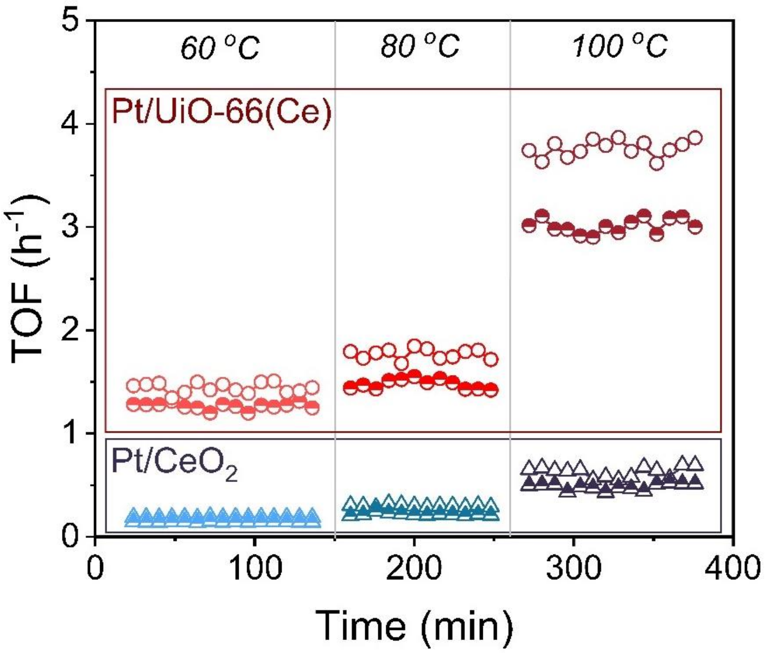

Figure S17. Calculated TOFs for the CO oxidation using Pt/UiO-66(Ce) (circles) and Pt/*n*CeO_2 materials (triangles) at different reaction temperatures and the following reaction conditions (Half-filled symbols: GHSV = 10000 mL/g_{cat}·h, 0.6% CO, 9.6% O_2; Empty symbols: GHSV = 22000 mL/g_{cat}·h, 0.08% CO, 4.0% O_2)

2.2. Comparison of the catalytic performance for the Pt/UiO-66(Ce) SAC (this work) with other state-of-the-art single-atom MOF-based representative works.

Table S3. Comparison of the catalytic performance for the Pt/UiO-66(Ce) (this work) with other cationic single atom MOF-based catalysts.

**Catalysts**	**CO Oxidation Conditions**	**Metal loading (%wt)**	**T (ºC)**	**TOF x 10^{-3} (s^{-1})**	**References**
Pt/UiO-66(Ce)	50 mL/min (0.6% CO, 9.6% O_2)<br>150 mg catalyst - 20000 mL/g_{cat}·h	0.07% Pt	80	0.42	This work
Pt/UiO-66(Ce)	50 mL/min (0.6% CO, 9.6% O_2)<br>150 mg catalyst - 20000 mL/g_{cat}·h	0.07% Pt	100	0.83	This work
Cu/UiO-66(Zr)	30 mL/min (1.0% CO, 1.0% O_2)<br>120 mg catalyst - 15000 mL/g_{cat}·h	3.0% Cu	80	0.22	1
Cu/MOF-808(Ce)	32.5 mL/min (1.0% CO, 2.5% O_2)<br>25 mg catalyst - 78000 mL/g_{cat}·h	10.0% Cu	100	0.11	2
Cu_1/UiO-66(Zr)	30 mL/min (1.0% CO, 15% O_2)<br>30 mg catalyst - 30000 mL/g_{cat}·h	2.0% Cu	120	0.95	3

2.3. Arrhenius plots

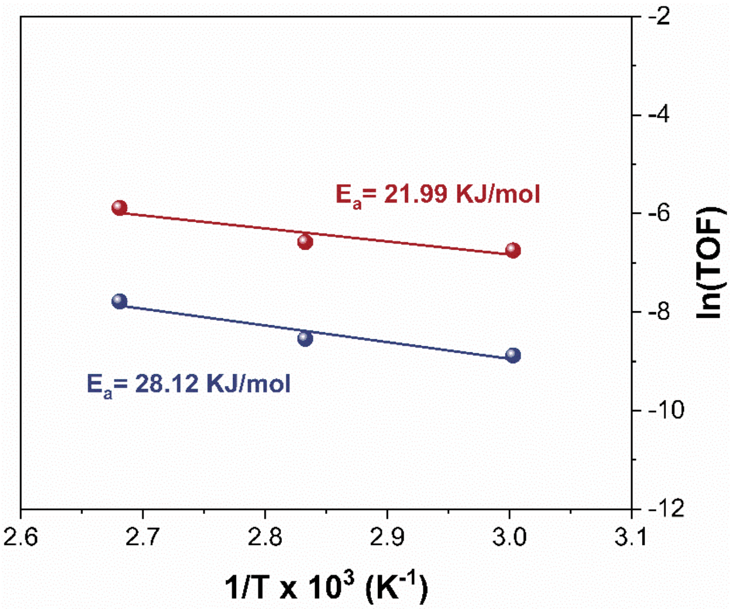

Figure S18. Arrhenius plots and apparent activation energy calculations for Pt/UiO-66(Ce) (red line) and Pt/*n*CeO_2 (blue line) at GHSV = 20000 mL/g_{cat}·h.

2.4. Pt/UiO-66(Ce) characterization after catalysis

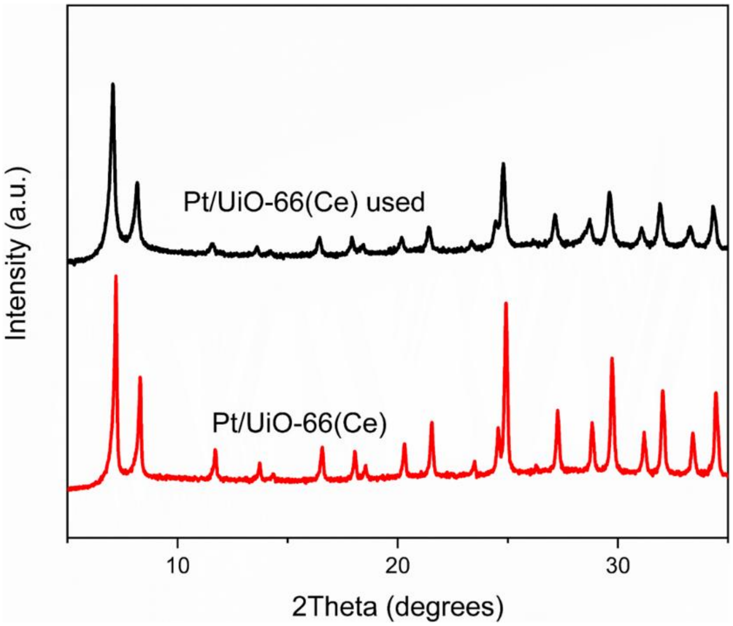

Figure S19. PXRD patterns of the as-synthesized Pt/UiO-66(Ce) (red line) and after the CO oxidation reaction (black line).

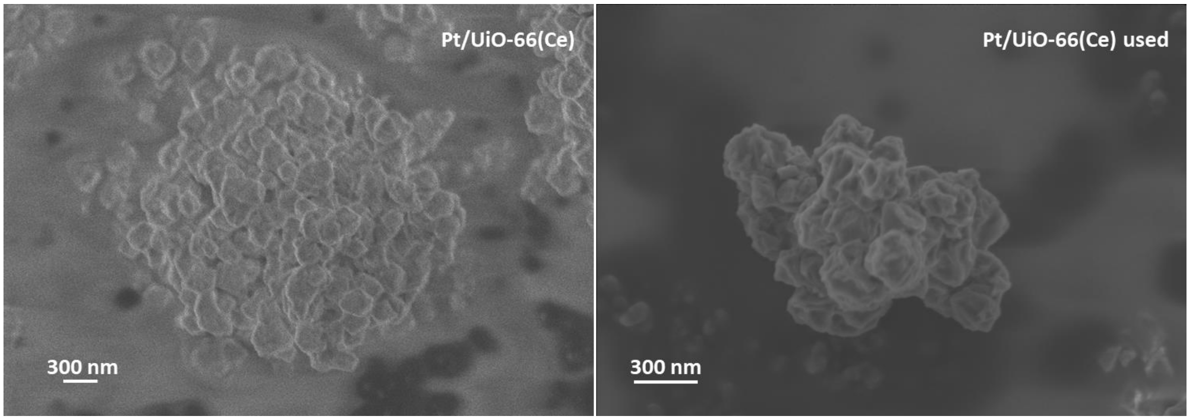

Figure S20. FESEM images of the Pt/UiO-66(Ce) (left) and after the CO oxidation reaction (right).

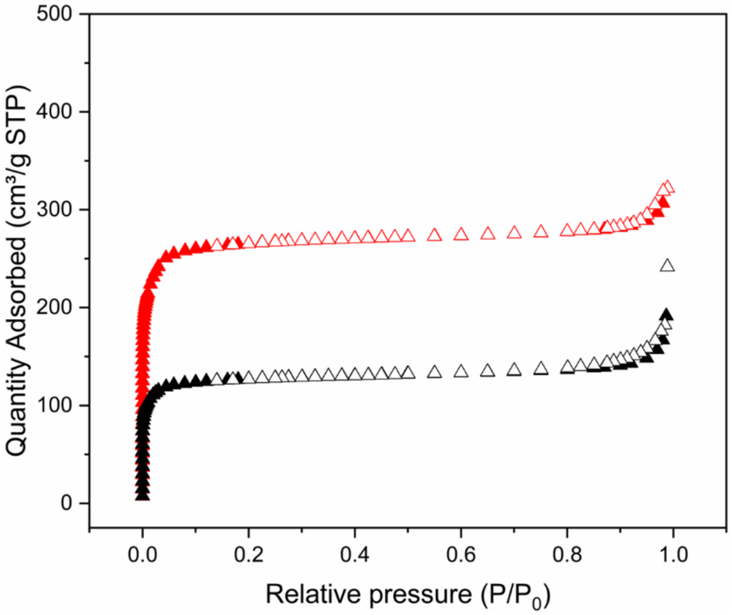

Figure S21. N_2 adsorption-desorption isotherms of the Pt/UiO-66(Ce) (as-synthesized: red triangles; after the CO oxidation: black triangles).

Table S4. N_2 adsorption isotherm report of fresh and used MOF catalyst, respectively.

Sample	BET surf. area (m^2/g)	Micro. area (m^2/g)	Micro. volume (cm^3/g)
Pt/UiO-66(Ce)	1071	1042	0.40
Pt/UiO-66(Ce) used	506	485	0.19

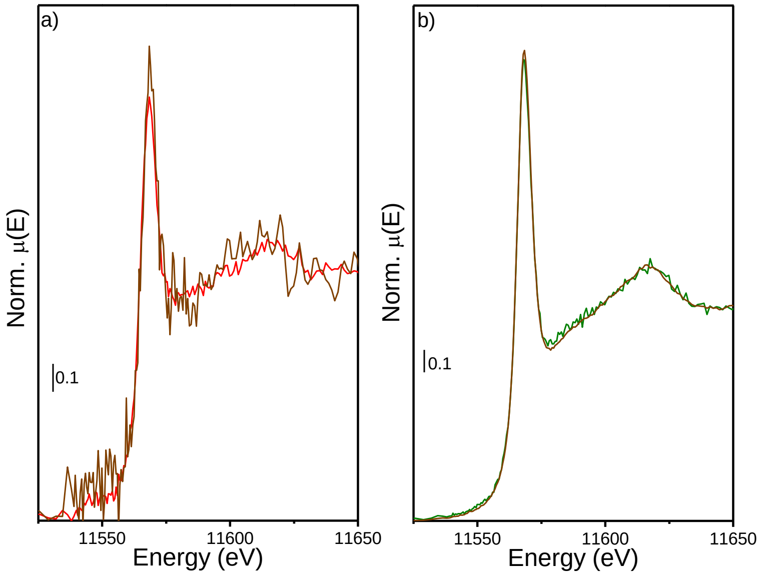

Figure S22. Pt L_3-XANES spectra of a) Pt/UiO-66(Ce) activated (red line) and after catalytic test at 100°C (brown line) and b) Pt/*n*CeO_2 as prepared (green line) and after catalytic test at 100°C (brown line).

3. References

(1) Abdel-Mageed, A. M.; Rungtaweevoranit, B.; Parlinska-Wojtan, M.; Pei, X.; Yaghi, O. M.; Behm, R. J. Highly Active and Stable Single-Atom Cu Catalysts Supported by a Metal–Organic Framework. *J. Am. Chem. Soc.* **2019**, *141* (13), 5201–5210.

(2) He, X.; Looker, B. G.; Dinh, K. T.; Stubbs, A. W.; Chen, T.; Meyer, R. J.; Serna, P.; Román-Leshkov, Y.; Lancaster, K. M.; Dincă, M. Cerium(IV) Enhances the Catalytic Oxidation Activity of Single-Site Cu Active Sites in MOFs. *ACS Catal.* **2020**, *10* (14), 7820–7825.

(3) Abdel-Mageed, A. M.; Rungtaweevoranit, B.; Impeng, S; Bansmann, J.; Rabeah, J.; Chen, S.; Häring, T.; Namuangrak, S.; Faungnawakij, K.; Brückner, A.; Behm, R. J. Unveiling the CO oxidation Mechanism over a Molecularly Defined Copper Single-Atom Catalyst Supported on a Metal-Organic Framework. *Angew. Chemie* 2023, e202301920.
